# Cutaneous mastocytosis in childhood 

**DOI:** 10.5414/ALX02304E

**Published:** 2022-01-05

**Authors:** Katja Nemat, Susanne Abraham

**Affiliations:** 1 Praxis für Kinderpneumologie/Allergologie am Kinderzentrum Dresden (Kid),; 2 Interdisziplinäre Pädiatrisch-dermatologische Sprechstunde, Universitäts AllergieCentrum (UAC) Dresden, and; 3 Klinik für Dermatologie, Universitätsklinikum Carl Gustav Carus Dresden, Germany

**Keywords:** mastocytosis, urticaria pigmentosa, mast cell, mast cell tryptase, antihistamines, anaphylaxis

## Abstract

Mastocytoses are characterized by clonal proliferation of mast cells in various tissues. In childhood, cutaneous mastocytosis (CM) occurs almost exclusively. It is confined to the skin, and has a good prognosis. The most common form is the maculopapular cutaneous mastocytosis (MPCM), formerly called urticaria pigmentosa. A distinction is made between a monomorphic variant of MPCM with multiple small, roundish maculopapular skin lesions and the – more common – polymorphic variant with larger lesions of variable size. One quarter of CM diagnosed in childhood are mastocytomas, which often occur solitary or at multiple sites. The diffuse variant of CM (DCM), which affects 5% of children with CM, should be distinguished from these forms. Systemic mastocytoses (SM) with mast cell infiltrates in the bone marrow or other extracutaneous tissues, such as the gastrointestinal tract, occur predominantly in adults. The diagnosis of CM is usually made clinically: Manifestation in infancy, typical morphology and distribution, pathognomonic Darier sign. Basal serum tryptase is determined if DCM or systemic mastocytosis are to be diagnosed. Children with mastocytosis should be managed in a specialized outpatient clinic. For affected families, detailed information about the clinical picture including prognosis assessment is essential. Mast cell mediated symptoms are controlled by oral non-sedating antihistamines if needed.

## Introduction 

**Figure 1:**
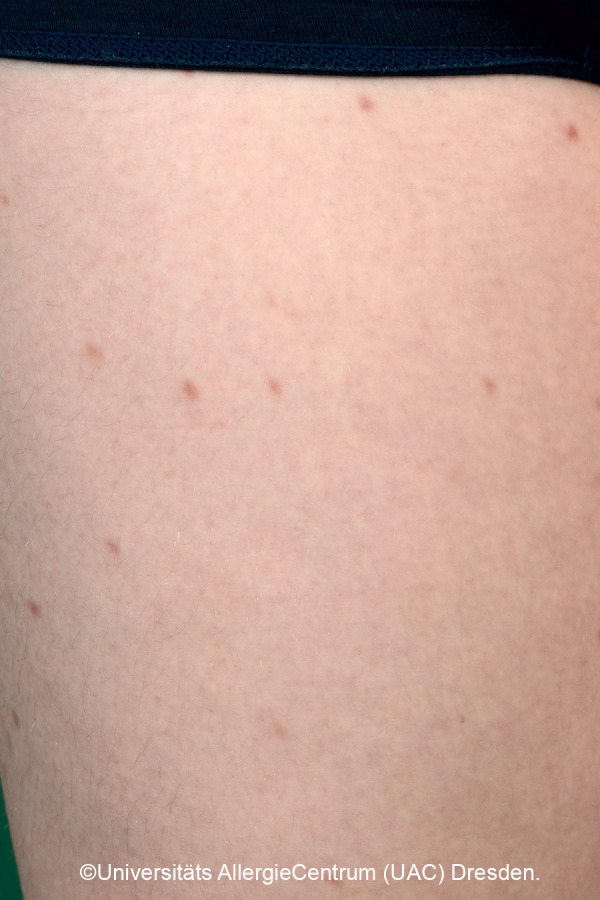
Monomorphic MPCM. Manifestation at approximately 6 – 10 years of age.

**Figure 2:**
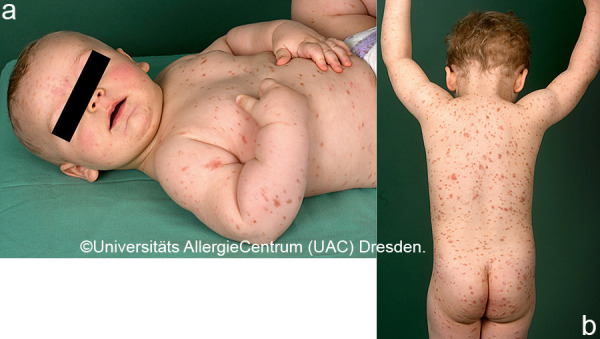
Polymorphic MPCM, baby (2a) and toddler (2b).

Mastocytoses are a heterogeneous group of diseases that are characterized by an accumulation of clonal mast cells in tissue. A distinction is made between cutaneous mastocytoses, which exclusively affect the skin, and systemic forms in which the bone marrow and/or other extracutaneous tissue are additionally affected (see classification, Table 1). In childhood, mastocytoses are almost exclusively cutaneous (cutaneous mastocytosis (CM)) [1, 3]. The most frequent form is maculopapular cutaneous mastocytosis (MPCM), formerly called “urticaria pigmentosa” (~ 70% of childhood CM) [1, 4]. This form manifests with yellow-brownish or red-brown papules and macules; their number can vary between < 10 and > 100. A distinction is made here between a monomorphic variant with multiple small, round maculopapular skin lesions (Figure 1) [1] and the – more common – polymorphic variant with slightly larger lesions of different configuration (Figure 2a, b). The polymorphic variant usually manifests in the first few weeks or half year of life, whereas the monomorphic variant, which most often affects the truncal area, only appears at toddler or even school age. 

**Figure 3:**
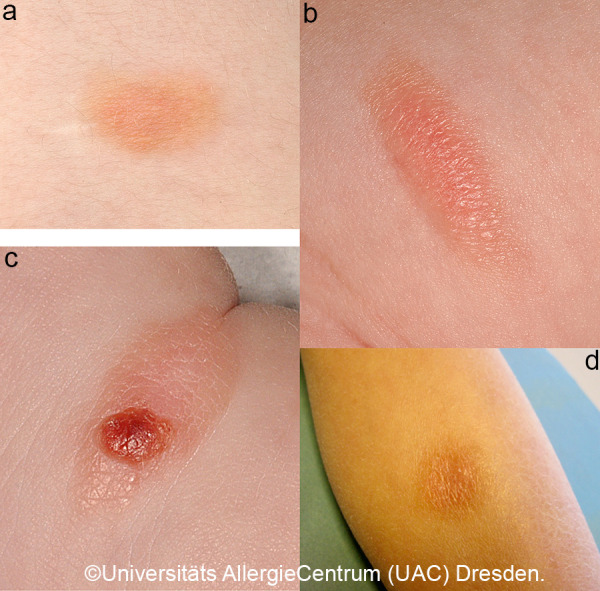
Solitay mastocytoma in an approximately 9-year-old child; c: with erosion; d: in healing.

**Figure 4:**
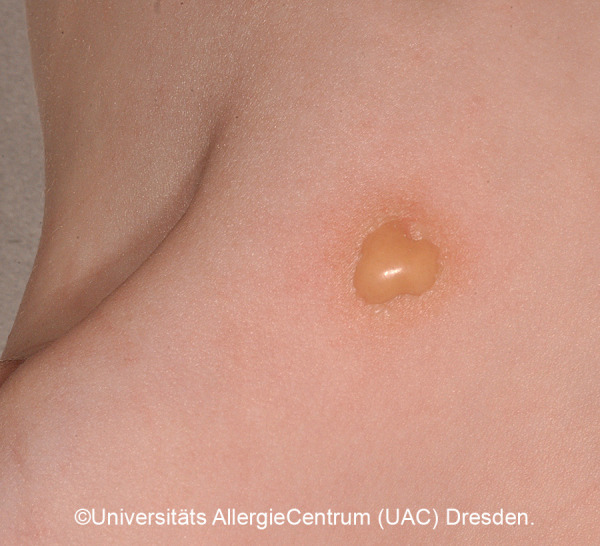
Darier’s sign with blistering.

**Figure 5:**
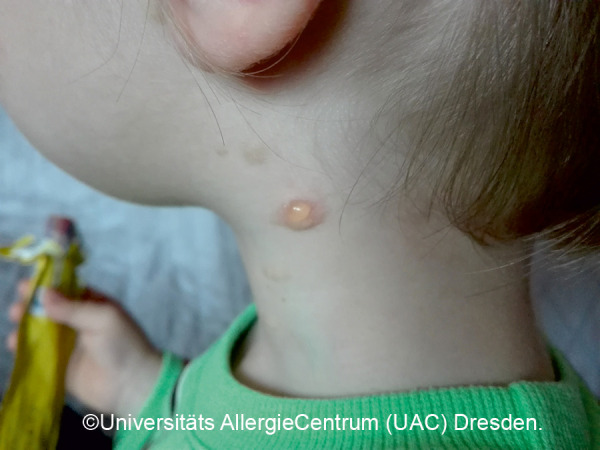
Typical finding on neck region; blistering.

A quarter of the CM diagnosed in childhood are mastocytomas, which often appear solitary or in different places (Figure 3a, b). Usually the yellow-brownish papules or plaques with a leather-like structure are noticeable in the first months of life; occasionally, blistering and consecutive erosion are most prominent (Figures 3c, Figure 4, Figure 5) [1, 4]. 

**Figure 6:**
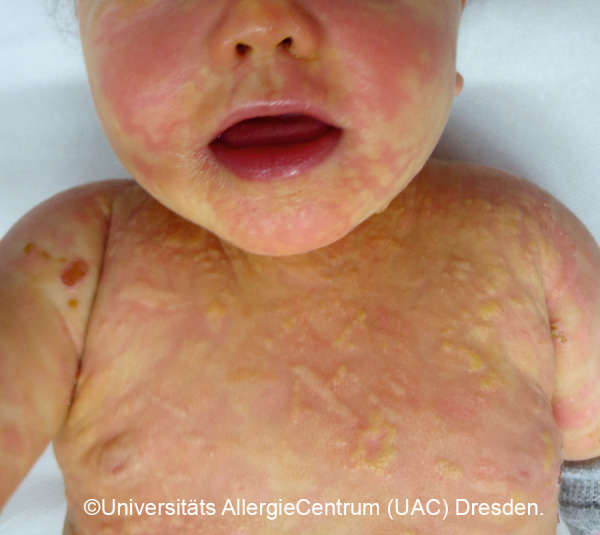
Diffuse cutaneous mastocytosis. (With permission of Prof. Peter Höger, Hamburg).

The diffuse variant of CM (DCM), which affects 5% of children with CM, must be distinguished from these forms [1]. It manifests early, in the first few months of life, sometimes even congenitally. The characteristic features are leather-like, thickened skin or edematous “orange-peel” skin; erythema or flushing frequently occur, and sometimes there is pronounced (often hemorrhagic) blistering (Figure 6). 

Systemic mastocytoses most frequently occur in adults. Here, mast cell infiltrates are found in the bone marrow or other extracutaneous tissue, e.g., the gastrointestinal tract. The skin is also frequently affected. In view of the sometimes existing association with hematological neoplasms, regular check-ups including ultrasound of the abdomen, colonoscopy, and bone marrow aspiration are necessary. In the case of systemic mastocytoses, depending on the organs involved, extracutaneous symptoms occur more frequently and there is a higher risk of anaphylactic reactions [5, 6]. 

This article presents the cutaneous forms of mastocytosis in childhood and adolescence. 

## Etiology 

Mastocytoses result from the clonal proliferation of mast cells in various tissues. This excessive mast cell proliferation is mainly caused by a genetic aberration, with adults and children having been shown to differ in terms of mutations in the KIT gene [3] 

In pediatric cutaneous mastocytosis, mast cell accumulations are only found on the skin, and the KIT mutation plays a subordinate role with regard to further clinical management. 

The KIT D816V point mutation is of particular relevance; it is detectable in ~ 90% of adults with systemic mastocytosis, as compared to 35% of patients with pediatric mastocytosis. In childhood mastocytosis, KIT mutations are found more frequently in other loci (exons 8, 9, 11); in up to 25%, no KIT mutations (KIT wild-type) are detectable at all [1, 7, 8, 9]. In contrast to adults with mastocytosis, the determination of the peripheral c-KIT mutation does not contribute to diagnosis and is therefore not necessary [7]. 

## Diagnosis 

Exact epidemiological information on mastocytosis is lacking; in any case, it is a very rare disease [10]. Adults with mastocytosis usually suffer from a systemic form. Cutaneous mastocytosis is frequently diagnosed before the second birthday, often even in the first 6 months of life [8, 11]. Thereafter, the incidence decreases until school age and rises again from age > 15 years [12]. 

**Figure 7:**
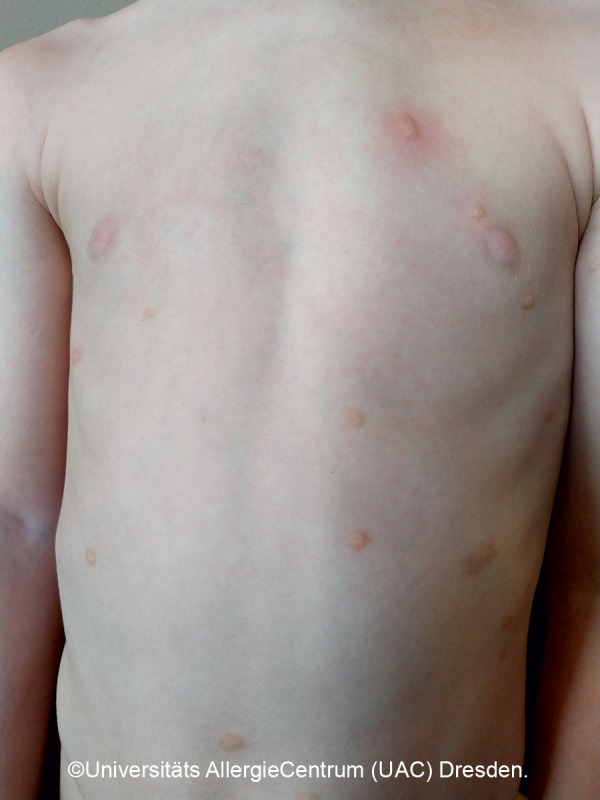
Several mastocytomas. Darier’s sign positive.

**Figure 8:**
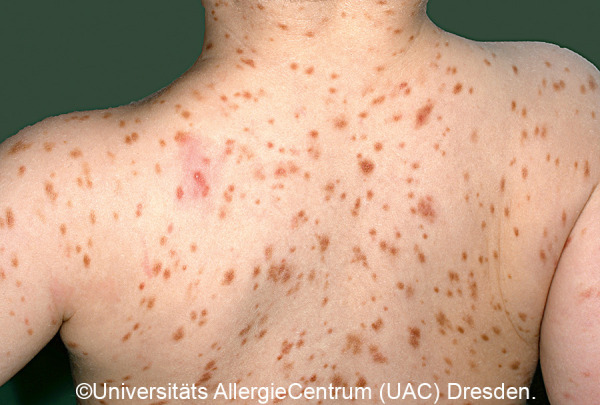
Polymorphic MPCM, toddler, Darier’s sign positive (left shoulder).

**Figure 9:**
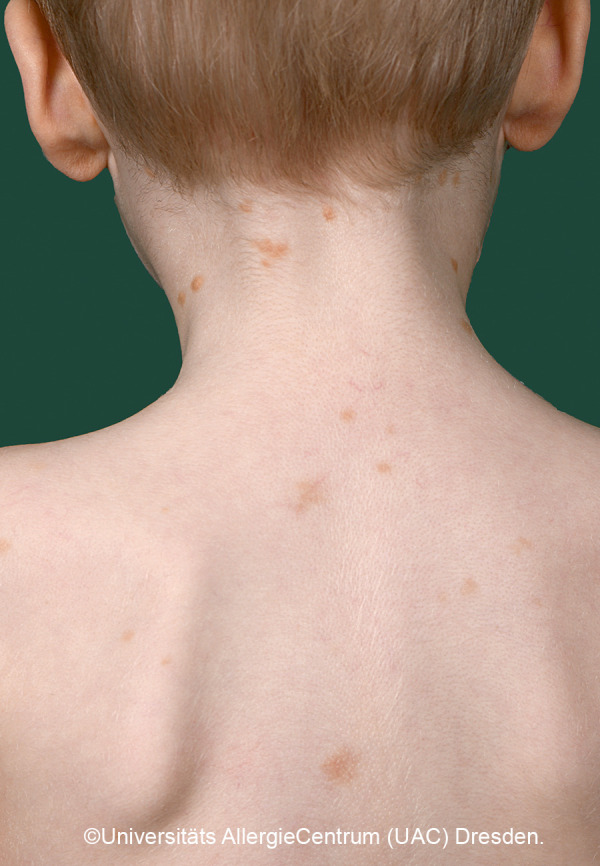
Polymorphic MPCM, typical pattern.

**Figure 10:**
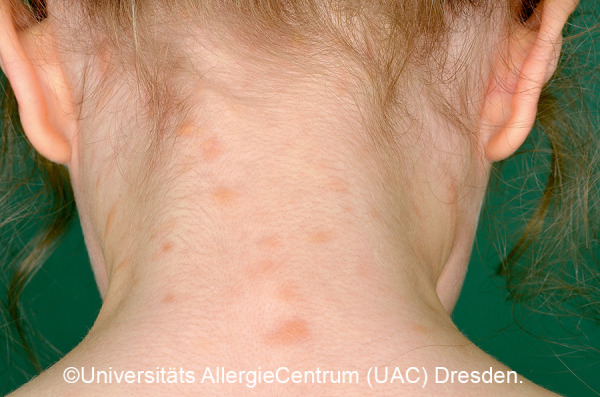
Polymorphic MPCM, typical pattern and localization with possible mechanical irritation.

Diagnosis of pediatric CM is usually made clinically based on age as well as typical morphology and distribution of the skin lesions (Table 1) (Figures 2a, 2b, Figure 5, Figure 7, Figure 8, Figure 9, Figure 10) and most often positive (pathognomonic) Darier’s sign (Figures 4, Figure 7, Figure 8). This means that mechanical irritation of the lesions, e.g., with a wooden spatula, leads to swelling and erythema within a few minutes. In young infants as well as when pronounced skin involvement or suspicion of DCM is present (Figure 6), Darier’s sign should be checked with caution, since a flush and even hypotension can be induced due to massive mast cell activation [1, 13]. Dermographic urticaria is sometimes present in monomorphic MPCM (Figure 1) and most often present in DCM (Figure 6). 

In unclear cases – especially if the clinical findings are inconclusive – a biopsy of the lesional skin can be performed to confirm the diagnosis. Hematoxylin-eosin staining can detect mast cell accumulations histologically. In most cases, however, no histological confirmation is necessary because the clinical picture is clear. 

The suspicion of systemic involvement or DCM can be confirmed by determination of the basal mast cell tryptase. Values above the 95^th^ percentile (11.4 ng/mL) are considered to be elevated. If serum tryptase is > 20 ng/mL in adults, further diagnostic workup should be considered. Normal ranges for younger children have not been well evaluated. In infants, toddlers, and schoolchildren without further warning symptoms (e.g., hepatomegaly, failure to thrive), usually no systemic mastocytosis is present, even if the serum tryptase level is increased. Therefore, in these cases, bone marrow aspiration, which is the standard diagnostic procedure in adults, is very rarely required in children. 

## Symptoms 

Symptoms in childhood CM are most often due to the effect of mast cell mediators (histamine, eicosanoids, cytokines, vasoactive substances, etc.) after degranulation. However, most children with CM present to a doctor for the first time because of their skin lesions and at this point do not show any other symptoms. 

In infants with MPCM who are affected as early as their first few months of life, attacks of flushing are sometimes observed, especially when heavily infiltrated lesions or pronounced skin involvement is present; in rare cases, even changes in consciousness due to arterial hypotension occur [4]. 

In solitary mastocytomas or polymorphic CM with plaques or nodular skin lesions, blistering occasionally occurs in the first few months until the first few years of life, especially when there is mechanical irritation (Figures 4, Figure 5). This is usually harmless and heals without scarring. 

DCM, in which larger skin areas are involved and a higher degree of infiltration is seen, presents with pronounced urticarial dermographism of the thickened skin, particularly in infants and toddlers (Figure 6). Blistering often occurs after slight irritation, and pronounced hemorrhagic bullous exacerbation also occurs. Serum tryptase is regularly, and significantly, increased in DCM, in contrast to the other cutaneous forms of the disease. Nevertheless, involvement of the bone marrow or extracutaneous tissue is not present in most cases. However, hepatomegaly should be excluded clinically and/or sonographically, as it is considered a predictor of systemic involvement. 

The largest review of childhood mastocytoses analyzed 1,747 case reports over a period of 54 years [8]. Méni et al. [8] show that the most common symptom was pruritus – 48% of cases (not stratified according to the different variants). Blistering was reported in a third of the children, flushing in 25%, gastrointestinal symptoms (abdominal pain, diarrhea, nausea) in 20%, and anaphylaxis in 5%. In other cohorts, similar, but sometimes also lower, symptom rates were observed [13, 18]. In particular, the occurrence of anaphylactic reactions in pediatric CM varies between 0 and 9% in the literature [13, 19]. Isolated respiratory symptoms such as rhinorrhea or bronchoconstriction mediated by mast cell mediators are rarely described in children with CM [16]. 

In general, the following risk factors for mast cell activation and anaphylaxis in pediatric CM can be identified: increased serum tryptase; presence of diffuse CM; excessively widespread skin lesions [13, 20, 21]. For the other forms, especially for uncomplicated polymorphic MPCM of moderate severity, the risk of anaphylaxis does not seem to be significantly increased compared to the general population (review by Brockow et al. 2021) [13]. In adults with mastocytosis, on the other hand, the risk of anaphylaxis is highly increased (incidence ~ 50%) [5, 22]. Interestingly, also the triggers of anaphylaxis in childhood CM differ significantly from those in adults with mastocytosis: in two thirds of cases, no trigger can be identified (idiopathic anaphylaxis) [21]. Food-allergic reactions play a role in 10 – 20% of cases, whereas drugs only play a role in < 10%, and insect bites do not seem to be any major trigger of anaphylactic reactions (main trigger in adults with mastocytosis) [5, 6, 21]. 

## Treatment/management 

Treatment should aim to control the mast cell-mediated symptoms, which is why histamine receptor antagonists are a central component of treatment [23]. Non-sedating antihistamines should primarily be used in this context. If symptoms persist, the administration of non-sedating H_1_ antihistamines can be increased to 4 times the daily dose, analogous to the treatment of chronic spontaneous urticaria [24]. 

If the symptoms still persist, the additional administration of H_2_ antihistamines and leukotriene antagonists, such as montelukast, can be considered. 

In a controlled clinical study using the H_1_ blocker rupatadine in mastocytosis, symptom control and an improvement in quality of life were demonstrated in adults [26]. A controlled short-term administration of sedating antihistamines is only justified when sleep problems are most prominent [27]. In the case of gastrointestinal symptoms, H_2_ blockers or proton pump inhibitors (previously also cromoglicic acid) are also used [25, 28]. 

For recurrent pruritus in solitary mastocytomas, the local application of a potent topical corticosteroid can be helpful; this can reduce the symptoms and improve the cosmetic issues [23, 25]. 

Topical calcineurin inhibitors such as pimecrolimus or tacrolimus can also reduce the activity of mechanically irritable and thus impairing mastocytomas [29, 30]. In individual cases, if Darier’s sign can be triggered easily and irritability is present in everyday situations, it makes sense to cover the lesion with a plaster bandage or protective clothing. Surgical excision should only be discussed in very rare situations, such as severely irritable solitary mastocytomas in an unfavorable location or induction of anaphylaxis by singular, heavily infiltrated mastocytomas. 

Since the pruritus is triggered by increased IL-31 levels [31] and nemolizumab, a specific IL-31 antibody, is currently being developed, its therapeutic use in severe forms of CM would be conceivable if this drug becomes available in the future [32]. 

Even if phototherapy with UVA1 light, narrowband UVB, or PUVA therapy can improve the skin condition, it should only be used in individual cases due to the potential carcinogenic risk in the mostly self-limiting course of CM in childhood [33]. 

The most important measure in the therapeutic management of children with mastocytosis is detailed information about the clinical picture, the harmlessness of the skin symptoms and the favorable prognosis that can be assumed in most cases. The first consultation should take place shortly after the diagnosis has been made. Otherwise, there will be a relatively high risk that families receive worrying misinformation from the internet or from medical staff who are not familiar with childhood mastocytosis. This is one of the reasons why it is recommended that patients consult a specialist early. Receiving a document that confirms the diagnosis and names contact persons for queries is also relieving. The prophylactic prescription of oral non-sedating antihistamines, such as cetirizine, in children who have not yet shown any symptoms as on-demand medication can also be seen in this context. If urticaria, flushing, or blistering were already observed, an oral antihistamine should always be used and instructions on how to deal with symptoms should be given. In cases of pronounced mediator-associated symptoms and severe impairment, a rectal or oral corticosteroid can also be used additionally for a short time. In individual cases of severe CM, omalizumab has also been reported to have a positive effect in children [34]. If risk factors for anaphylactic reactions have been identified, the family must be provided with an emergency kit including an adrenaline auto-injector [35]. In these cases, anaphylaxis training is also indicated**.**

### Vaccinations and mastocytosis 

In the literature, a slightly higher risk of undesirable side effects after the first vaccination for children with mastocytosis is discussed. For booster vaccinations, no recurrence risk is described, and the reactions were mild, with symptoms attributable to mast cell degranulation. In individual cases, e.g., those with pronounced skin involvement, bullous or flush reactions or with DCM, 2-hour clinical monitoring after the first vaccination should be considered. In general, children with mastocytosis should receive the regular vaccinations as recommended by the national vaccine advisory commission. Childhood mastocytosis is often only diagnosed after the start of the vaccinations. The patients of our pediatric dermatological mastocytosis clinic did not retrospectively report clinical reactions to vaccinations. For adults and adolescents with mastocytosis, expert recommendations on risk stratification and monitoring period for COVID-19 vaccinations have recently been published. The authors did not formulate a contraindication due to mastocytosis. The recommendations have not been evaluated so far [39]. 

## Prognosis 

Prognosis of childhood mastocytosis is generally good. If CM manifests in early childhood, spontaneous remission usually occurs in adolescence at the latest. To assume a prepubertal remission as regular, is too short-sighted in many cases [15]. The rate is ~ 70%. In most cases of polymorphic MPCM, clinical signs are no longer present in adulthood. The course over the years is characterized by a flattening of the initially elevated skin lesions (Figure 3d). Only pronounced forms have a prolonged course or reach only incomplete remission. Particularly, monomorphic MPCM that manifests late, from school age onwards, can develop into systemic forms, which is why monitoring of serum tryptase and, if necessary, staging can be helpful here [40, 41]. 

In diffuse CM, serum tryptase levels are usually elevated, although there is no bone marrow involvement or other systemic form. Nevertheless, the prognosis is favorable in those cases too, with extensive healing of the skin lesions and a decrease in serum tryptase by adolescence. Only a small proportion of DCM, especially familial forms due to c-kit germline mutations, develop into systemic forms of mastocytosis. A persistently high tryptase value, but also hepatomegaly or splenomegaly, can be an indicator for this [4]. 

The prognosis of (solitary) mastocytomas is excellent, even if a positive Darier’s sign (Figures 7, Figure 8) can often be detected for several years and local blistering often occurs initially (Figures 3c, Figure 4, Figure 5). Remission is achieved by adulthood, and there is no transition to systemic mastocytosis [12]. 

In view of the above-mentioned in most cases benign prognosis of pediatric mastocytoses, it is recommended that the affected children and adolescents consult a specialist. The families can be supported by offering regular, for example annual, visits. It can be discussed how to deal with mast cell mediator-associated symptoms, and individual risk factors can be identified at an early stage. Overall, in most cases it is possible to prevent serious disease-related impairment of the quality of life. 

## Funding 

None. 

## Conflict of interest 

The authors state that there is no conflict of interest. 

**Table 1.** Classification of mastocytoses (WHO classification, update 2016 and 2021 [42, 43]) and clinical signs/characteristics.

**Cutaneous mastocytoses (CM)**	*Maculopapular cutaneous mastocytosis* (MPCM) (formerly: *urticaria pigmentosa)*	*• Monomorphic variant:* Multiple round macules or papules, low degree of infiltration. Size: several millimeters to <1 cm. Localization: trunk, arms. Figure 1 *• Polymorphic variant:* Heterogeneous shape, limitation, and size; sometimes elevated. Size: several millimeters to several centimeters. Localization: disseminated on the trunk, neck, buttocks, groin, extremities; palmar and plantar skin typically not involved. Figures 2a, 2b, Figure 9, Figure 10	*• Darier’s sign:* pathognomonic for all forms (Figures 4, Figure 5, Figure 7, Figure 8) *• Serum tryptase*: - In *monomorphic MPCM* sometimes increased - In *polymorphic MPCM* and *mastozytomas* usually not increased. - In *DCM* usually highly increased. *• Histology*: multifocal or diffuse mast cell infiltration of the skin.
	Mastocytomas	*• 1 to 3* individual, slightly elevated, sharply demarcated, red-yellow-brown papules or plaques. Size: a few millimeters to centimeters; no preferred location. Figure 3a, 3b, 3c, 3d	
	*Diffuse cutaneous mastocytosis (DCM)*	• Large to generalized orange-peel-like, doughy, edematous yellow-brown-reddish skin lesions Systemic involvement possible. Figure 6	
Systemic mastocytoses (SM)	*Indolent mastocytosis (ISM)*	• Mast cell infiltration without organ dysfunction, not associated with hematologic neoplasms	Diagnostic criteria of SM *(1 major and 1 minor criterion or at least 3 minor criterion): * Major criteria: • Multifocal, dense MC infiltrations in bone marrow (≥ 15 adjacent MC) and/or other extracutaneous organs Minor criteria: • ≥ 25% atypical spindle-shaped MC in bone marrow or extracutaneous organs • KIT 816 point mutation in bone marrow or extracutaneous organs • Mast cells in bone marrow, peripheral blood, or other extracutaneous organs express CD25 and/or CD2 and/or CD 30 • Basal serum tryptase > 20 ng/mL
	*“Smoldering SM” (SSM)*	• High degree of infiltration in bone marrow (≥ 30%), organomegaly • Serum tryptase > 200 ng/mL	
	*SM with associated hematological neoplasm (SM-AHM)*	• SM with clonal hematologic disease	
	*Aggressive SM (ASM)*	• Mast cell infiltration with organ dysfunction, without mast cell leukemia	
	*Mast cell leucemia (MCL)*	• Diffuse infiltration of atypical mast cells in the bone marrow: ≥ 20% in bone marrow smear, ≥ 10% in the peripheral blood	
**Mast cell sarcoma**		• Solid tumor made up of atypical mast cells • Extremely rare
